# Socio-economic inequalities in cancer survival: how do they translate into Number of Life-Years Lost?

**DOI:** 10.1038/s41416-022-01720-x

**Published:** 2022-02-11

**Authors:** Aimilia Exarchakou, Dimitra-Kleio Kipourou, Aurélien Belot, Bernard Rachet

**Affiliations:** Inequalities in Cancer Outcomes Network (ICON), Department of Non-Communicable Disease Epidemiology, Faculty of Epidemiology and Population Health, London School of Hygiene and Tropical Medicine, Keppel Street, London, WC1E 7HT UK

**Keywords:** Cancer epidemiology, Health policy, Prognosis, Epidemiology, Cancer

## Abstract

**Background:**

We aimed to investigate the impact of socio-economic inequalities in cancer survival in England on the Number of Life-Years Lost (NLYL) due to cancer.

**Methods:**

We analysed 1.2 million patients diagnosed with one of the 23 most common cancers (92.3% of all incident cancers in England) between 2010 and 2014. Socio-economic deprivation of patients was based on the income domain of the English Index of Deprivation. We estimated the NLYL due to cancer within 3 years since diagnosis for each cancer and stratified by sex, age and deprivation, using a non-parametric approach. The relative survival framework enables us to disentangle death from cancer and death from other causes without the information on the cause of death.

**Results:**

The largest socio-economic inequalities were seen mostly in adults <45 years with poor-prognosis cancers. In this age group, the most deprived patients with lung, pancreatic and oesophageal cancer lost up to 6 additional months within 3 years since diagnosis than the least deprived. For most moderate/good prognosis cancers, the socio-economic inequalities widened with age.

**Conclusions:**

More deprived patients and particularly the young with more lethal cancers, lose systematically more life-years than the less deprived. To reduce these inequalities, cancer policies should systematically encompass the inequities component.

## Background

Patients living in more socioeconomically deprived areas (referred hereafter as ‘more deprived’ patients) tend to have worse cancer outcomes than those living in less deprived areas (‘less deprived’ patients), in the UK and other countries [1–4]. In England, in order to improve cancer survival and reduce the inequalities, the first-ever NHS Cancer Plan was implemented in 2000, followed by several successive policy initiatives, mainly focusing on promoting early diagnosis, optimising treatment pathways and maximising available resources to bring better treatment options, care and infrastructure [5–9]. However, the indisputable overall increase in cancer survival over the last 25 years has been accompanied by a minimal or lack of improvement in socio-economic inequalities, reflected on persistent poorer cancer prognosis of the more deprived patients [10]. Similar patterns have been repeatedly reported regarding cancer screening uptake [11, 12] and vaccine coverage [13–17]. Such inequalities pose a challenge for the National Health Service (NHS) which is committed to equity of access in healthcare, i.e. equal access for equal need for the whole population.

Research has shown that cancer awareness, clinical (comorbidities) and tumour-related (tumour stage) factors can only explain part of the inequalities in England [18–20] and that more emphasis should be given to the observed variation in cancer screening uptake [21–23] and management of patients [24–26]. However, communication of these epidemiological findings with political forces and stakeholders has been suboptimal, evidenced by the lack of initiative to target inequalities in a more methodical fashion.

Socio-economic inequalities in England have been described previously through survival or mortality probabilities [4, 10, 27]. Although these measures are necessary for evaluating the patients’ prognosis, they do not fully reflect the burden on the society, unlike alternative measures such as the crude probability of death from cancer (CPr) [28] or the Number of Life-Years Lost (NLYL) due to cancer [29, 30]. The NLYL measures how many years patients diagnosed with cancer can lose due to their cancer. The measure, easy to communicate to a large audience [29], can also be translated into societal or economic cost.

This study aims to quantify the population burden of socio-economic inequalities (measured with the income deprivation domain of the Index of Multiple Deprivation for a given area) in cancer survival using the CPr and NLYL due to cancer, to identify specific components for improvement, and to consider how this can be integrated with public health policy and resource allocation.

## Methods

### England National Cancer Registry data

The main source of data was the population-based National Cancer Registry of England. We included all patients aged 15–99 years, diagnosed with a primary, invasive, malignant (ICD-O behaviour code 3) neoplasm between 1 January 2010 and 31 December 2014 and followed up to 31 December 2015. The tumour site was coded according to the tenth revision of the International Classification of Diseases (ICD-10) [31] while the second edition of the International Classification of Diseases for Oncology (ICD-O-2) was used for morphology and behaviour [32]. We included 23 of the most common cancers in males and females.

Socio-economic deprivation of patients was based on the income domain of the English Index of Multiple Deprivation (IMD 2004) [33], an ecological measure of relative deprivation. The income domain score measures the proportion of the population with low income living in a given Lower layer Super Output Area (LSOA) [34]. LSOAs are census-based administrative spatial areas developed by the Office for National Statistics (ONS) and designed for reporting small area statistics in England and Wales. Cancer patients were assigned to their LSOA of residence at diagnosis (32,482 LSOAs in England, mean population 1500). They were allocated to a deprivation category (from 1, ‘least deprived’, to 5, ‘most deprived’) based on the quintiles of the national distribution of all LSOA-level income domain scores of the IMD 2004.

Among the seven domains of the IMD, we used the income domain firstly because of its overall high degree of agreement with the overall composite IMD measure [35]. Also, using the overall IMD can lead to misinterpretation because it contains components about access to public services, therefore access to optimal care, which is strongly linked to inequalities in cancer survival.

### Cancer survival measures

The estimation of cancer survival measures requires competing risks methods to account for the fact that cancer patients may die from causes other than the cancer under study [29, 36, 37]. However, as the cause of death is often unavailable or unreliable in population-based data, survival measures are estimated using methods from the relative survival framework. Assuming that the overall mortality hazard can be expressed as the sum of the cancer-related hazard (‘excess hazard’) and the hazard of death from other causes (‘expected hazard’), the basic principle in the relative survival framework is that the expected hazard is derived from the mortality hazard in the general population where patients come from, i.e. lifetables. The England lifetables are here defined by sex, age (0–99 by 1-year age groups), deprivation (1–5 using IMD) and for the calendar period 2010–2015 (by calendar year for 2010 and 2011, and assuming a plateau afterwards) and extracted from a dedicated website [38].

The NLYL can be estimated directly from the CPr, which is the probability of dying from cancer before or at time *t* in the presence of competing causes of death [39]. By integrating the CPr function from 0 to time *t* we can derive the NLYL which can be interpreted as the meantime patients would lose due to cancer death within a specific time period [0,*t*] [40, 41]. Although we provide a brief explanation in the Appendix, methods to estimate the CPr from a given cause in the relative survival framework have been fully described elsewhere [39–43]. NLYL is estimated in a pre-specified follow-up time window to account for the inability to estimate the entire survival function due to right-censoring.

We estimated the CPr and the NLYL due to cancer within 1 and 3 years after cancer diagnosis according to deprivation, age and sex. We present here the comparison of Life-Years Lost (LYL) within 3 years since diagnosis between the least and the most deprived patients. More detailed results (in particular for 1 year since diagnosis and all deprivation levels) are presented in the Supplementary file and the web-tool (https://CPr of death and NLYL due to cancer by deprivation/). Calculations were performed with R software version 4.0.4 and the package ‘relsurv’ version 2.2-3 [40]. To estimate 95% confidence intervals for the NLYL, we used the R-package ‘boot’ [44] version 1.3-28, for non-parametric bootstrap (1000 bootstrap replicates).

To describe the (cancer *j*)-specific burden among all different cancers combined in each group of patients defined by the combination of sex, age group and deprivation, we also present the proportion of NLYL due to each cancer over the total NLYL due to all cancers under study (k = 1,…, 23) for this group of patients. This quantity is weighted with the cancer-specific proportion of patients with each cancer over the total number of cancer patients in that group of patients. So, within a combination of sex/age/deprivation, this proportion can be expressed mathematically as follows:

$$P_j = \frac{{{{NLYL}}_j}}{{\mathop {\sum }\nolimits_k {{NLYL}}_k}} \cdot \frac{{n_j}}{{\mathop {\sum }\nolimits_k n_k}}$$

where j = 1,…, 23 defines the cancer and *n*_*j*_ the number of cases observed for that cancer and the specific subgroup studied.

## Results

During 2010–2014, more than 1.2 million patients were diagnosed with one of the 23 cancer sites in England, representing 92.3% of all incident cancers in England. Based on the area of residence at diagnosis, 20–21% of the patients were in each of the deprivation levels 1 (least deprived) to 4, contrasting with 17% in the most deprived group (level 5).

Among the most frequent cancers, colon, prostate and breast (female) cancers were more common in the less deprived whilst lung cancer largely predominated in the more deprived patients (Table 1). Cervical, stomach, liver and oesophageal cancers were more frequent in the more deprived than the less deprived patients. In contrast, pancreatic cancer was equally common in all deprivation groups.

**Table 1:** Number of cases and proportion of cancer patients in each deprivation level diagnosed with one of 23 cancer sites, 2010–2014.

	Deprivation					
	1 (least deprived) (*N* = 258,682)	2 (*N* = 264,762)	3 (*N* = 259,183)	4 (*N* = 249,154)	5 (most deprived) (*N* = 210,420)	Total (*N* = 1,242,201)
Cancer						
Bladder	8600 (3.3%)	9170 (3.5%)	9269 (3.6%)	8845 (3.6%)	7124 (3.4%)	43,008 (3.5%)
Brain	4186 (1.6%)	4288 (1.6%)	4011 (1.5%)	3684 (1.5%)	2922 (1.4%)	19,091 (1.5%)
Breast (female)	45,831 (17.7%)	44,486 (16.8%)	42,635 (16.4%)	38,982 (15.6%)	29,902 (14.2%)	201,836 (16.2%)
Cervix	1836 (0.7%)	2068 (0.8%)	2443 (0.9%)	2875 (1.2%)	3323 (1.6%)	12,545 (1.0%)
Colon	23,662 (9.1%)	24,065 (9.1%)	23,058 (8.9%)	21,279 (8.5%)	16,591 (7.9%)	108,655 (8.7%)
Hodgkin lymphoma	1416 (0.5%)	1514 (0.6%)	1491 (0.6%)	1683 (0.7%)	1629 (0.8%)	7733 (0.6%)
Kidney	8875 (3.4%)	9481 (3.6%)	9335 (3.6%)	9124 (3.7%)	7709 (3.7%)	44,524 (3.6%)
Larynx (male)	1046 (0.4%)	1310 (0.5%)	1400 (0.5%)	1854 (0.7%)	1969 (0.9%)	7579 (0.6%)
Leukaemia	7776 (3.0%)	7856 (3.0%)	7589 (2.9%)	6968 (2.8%)	5684 (2.7%)	35,873 (2.9%)
Liver	3393 (1.3%)	3603 (1.4%)	4022 (1.6%)	4232 (1.7%)	4498 (2.1%)	19,748 (1.6%)
Lung	24,773 (9.6%)	30,460 (11.5%)	34,973 (13.5%)	41,471 (16.6%)	43,959 (20.9%)	175,636 (14.1%)
Melanoma	15,587 (6.0%)	13,695 (5.2%)	12,099 (4.7%)	9646 (3.9%)	5709 (2.7%)	56,736 (4.6%)
Myeloma	4781 (1.8%)	4807 (1.8%)	4437 (1.7%)	4063 (1.6%)	3385 (1.6%)	21,473 (1.7%)
Non-Hodgkin lymphoma	11,874 (4.6%)	11,981 (4.5%)	11,367 (4.4%)	10,454 (4.2%)	8288 (3.9%)	53,964 (4.3%)
Oesophagus	6593 (2.5%)	7376 (2.8%)	7451 (2.9%)	7475 (3.0%)	6535 (3.1%)	35,430 (2.9%)
Ovary	6193 (2.4%)	6402 (2.4%)	6195 (2.4%)	5976 (2.4%)	4813 (2.3%)	29,579 (2.4%)
Pancreas	7535 (2.9%)	8048 (3.0%)	7906 (3.1%)	7550 (3.0%)	6186 (2.9%)	37,225 (3.0%)
Prostate	45,992 (17.8%)	43,920 (16.6%)	39,627 (15.3%)	33,438 (13.4%)	24,686 (11.7%)	187,663 (15.1%)
Rectum	12,135 (4.7%)	12,649 (4.8%)	12,106 (4.7%)	11,569 (4.6%)	9344 (4.4%)	57,803 (4.7%)
Stomach	4723 (1.8%)	5484 (2.1%)	5668 (2.2%)	6110 (2.5%)	5979 (2.8%)	27,964 (2.3%)
Testis	1755 (0.7%)	1836 (0.7%)	1962 (0.8%)	1952 (0.8%)	1819 (0.9%)	9324 (0.8%)
Thyroid	2726 (1.1%)	2520 (1.0%)	2469 (1.0%)	2606 (1.0%)	2444 (1.2%)	12,765 (1.0%)
Uterus	7394 (2.9%)	7743 (2.9%)	7670 (3.0%)	7318 (2.9%)	5922 (2.8%)	36,047 (2.9%)
Gender						
Male	134,648 (52.1%)	137,151 (51.8%)	132,468 (51.1%)	125,252 (50.3%)	106,557 (50.6%)	636,076 (51.2%)
Female	124,034 (47.9%)	127,611 (48.2%)	126,715 (48.9%)	123,902 (49.7%)	103,863 (49.4%)	606,125 (48.8%)
Age in years						
15–44	14,757 (5.7%)	15,130 (5.7%)	16,033 (6.2%)	17,669 (7.1%)	17,580 (8.4%)	81,169 (6.5%)
45–54	26,086 (10.1%)	24,998 (9.4%)	24,371 (9.4%)	25,282 (10.1%)	23,434 (11.1%)	124,171 (10.0%)
55–64	49,996 (19.3%)	49,648 (18.8%)	48,399 (18.7%)	47,080 (18.9%)	42,298 (20.1%)	237,421 (19.1%)
65 plus	167,843 (64.9%)	174,986 (66.1%)	170,380 (65.7%)	159,123 (63.9%)	127,108 (60.4%)	799,440 (64.4%)

### Number of Life-Years Lost due to the cancer

**Fig. 1:**
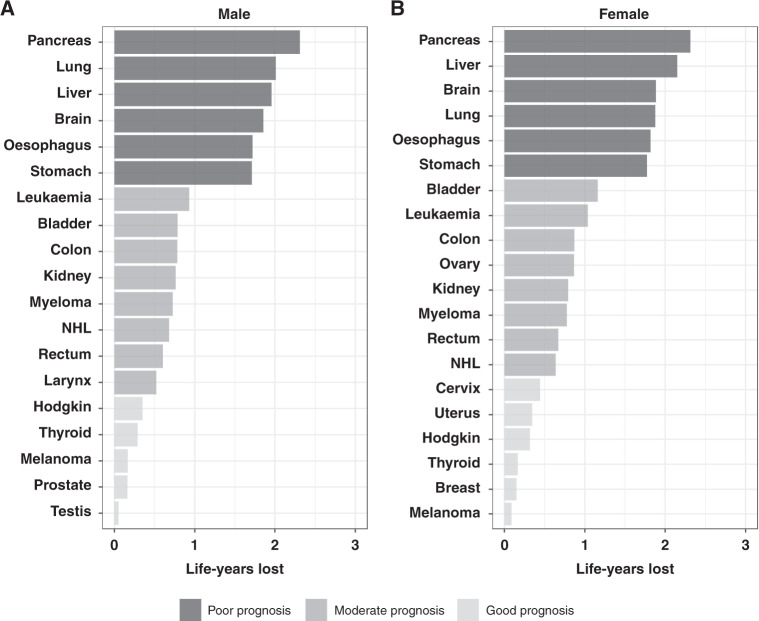
Number of life-years lost within 3 years due to a given cancer. **A** Male and **B** female patients diagnosed in 2010–2014. NHL non-hodgkin lymphoma.

The estimates of CPr and NLYL within 3 years divide naturally the cancer sites in ‘good’ (CPr: 0–0.25) or ‘moderate’ (CPr: 0.25–0.75) and ‘poor’ (CPr: 0.75–1) prognosis (Fig. 1; Supplementary Fig. 1). The cancer sites with the highest probability of death due to cancer within 3 years since diagnosis were brain, lung and all the upper-digestive organ cancers (pancreatic, liver, oesophagus and stomach) (Supplementary Fig. 1). For these cancers, the CPr within 3 years was between 0.75 and 1 and the NLYL within 3 years was between 1.75 and 2.3 years (Fig. 1).

Cancer sites with relatively low CPr within 3 years (<0.25) and NLYL of less than 0.5 years within 3 years, were Hodgkin lymphoma, thyroid, skin melanoma, female breast cancer and cancers of the reproductive organs, such as prostate and testicular cancer in male, and cervical and uterine cancers in female. The remaining cancers presented an intermediate CPr within 3 years (0.25–0.50), with 0.5–1.2 LYL within 3 years, and included the cancers of colon, rectum, kidney, bladder, larynx (men), ovary and leukaemia, myeloma and Non-Hodgkin lymphoma (NHL) (Fig. 1).

### Number of Life-Years Lost in different deprivation groups

**Fig. 2:**
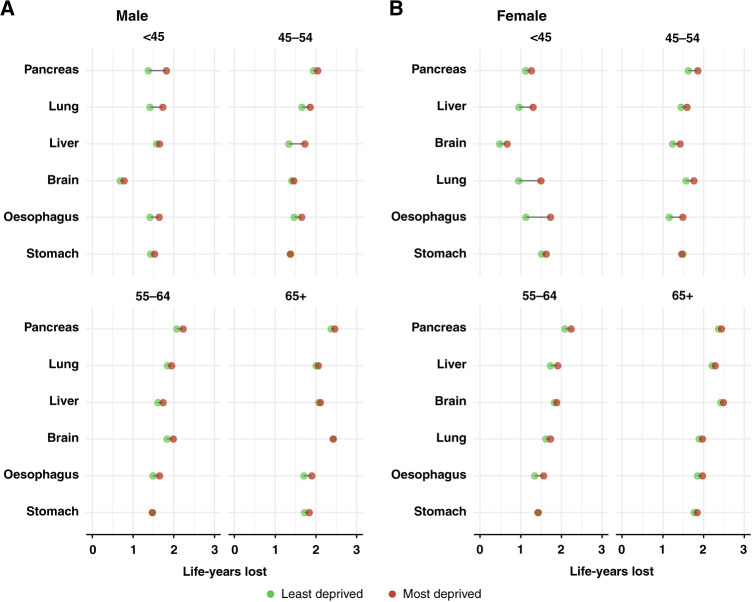
Number of life-years lost within 3 years due to a given cancer in the most and the least deprived by age group, for the group of poor-prognosis cancers. **A** Male, **B** female; cancers sorted as in Fig. 1.

**Fig. 3:**
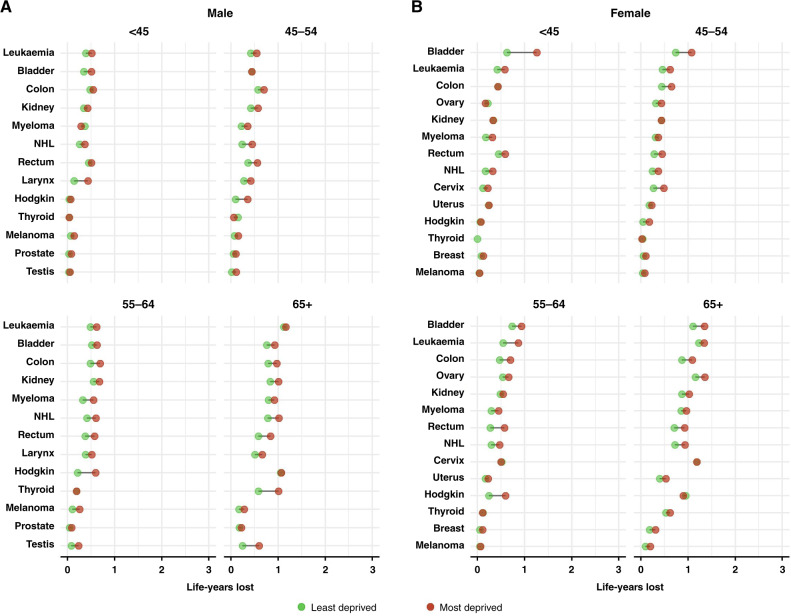
Number of life-years lost within 3 years due to a given cancer in the most and the least deprived by age group, for the group of moderate and good prognosis cancers. **A** Male, **B** female. NHL non-hodgkin lymphoma; cancers are sorted as in Fig. 1.

The NLYL within 3 years was consistently higher in the older age groups in both sexes (Figs. 2, 3), reflecting an overall worsening cancer prognosis with increasing age. Also, the most deprived patients had more LYL due to cancer than the least deprived for most of the cancer sites considered. However, the magnitude of the inequalities in the NLYL varied by sex and age group.

For the group of poor-prognosis cancers, the largest socio-economic inequalities were seen mostly in younger adults less than 45 years old. In particular, the most deprived male patients with pancreatic cancer lost 1.81 years within 3 years (95% CI: 1.56, 2.07) in contrast to the least deprived who lost 1.38 years (95% CI: 1.05, 1.71). Similarly, the most deprived female patients of less than 45 years old with lung cancer lost 1.49 years (95% CI: 1.34, 1.63), 0.54 years more than the least deprived (0.95; 95% CI: 0.77, 1.16) (Fig. 2; Supplementary Table 1). In contrast, an almost non-existent deprivation ‘gap’ was seen for brain cancer in (particularly male) patients more than 65 years old, with the NLYL within 3 years reaching nearly 2.5 years.

For the majority of the moderate and good-prognosis cancers (colon, rectum, kidney, leukaemia (female), myeloma (male), Non-Hodgkin lymphoma, testis, female breast, ovary, uterus), the difference in the NLYL between the most and least deprived mostly widened with age. For thyroid cancer, the deprivation difference peaked at 65 plus with no pattern in the other age groups. In contrast, the deprivation gap narrowed with age for bladder cancer in females and laryngeal cancer in males (Fig. 3; Supplementary Table 2; Supplementary Table 3).

One of the most striking socio-economic inequalities among all cancer-sex-age combinations for the moderate/good prognosis cancers was observed for Hodgkin lymphoma particularly in patients aged 55–64. In this age group, most deprived patients lost almost 0.4 additional years (within 3 years) compared to the least deprived in both male and female patients (Fig. 3; Supplementary Table 3) while no such wide inequalities were seen in the younger or the older age groups. In females, the largest difference was seen for bladder cancer in young women less than 45 years old, although deprivation differences -albeit smaller- were observed in most age groups. The NLYL in the most deprived women less than 45 years with bladder cancer was 1.26 years within three years (95% CI: 0.89, 1.65), 0.63 years more than the least deprived (NLYL = 0.63; 95% CI: 0.16, 1.15). In males, in addition to Hodgkin lymphoma, the deprivation difference was also particularly high for laryngeal cancer in adults less than 45 years and, thyroid and testicular cancer in the over 65 year olds (Fig. 3; Supplementary Table 2; Supplementary Table 3).

In contrast, the deprivation gap in the NLYL was small for skin melanoma in both male and female patients. Also, small variations between age groups and relatively small deprivation inequalities were seen for prostate cancer and for cervical and thyroid cancer in women. A reversal of the difference was observed for ovarian cancer in patients less than 45 years and Hodgkin lymphoma in female patients more than 65 years old.

### The proportion of Life-Years Lost

More life-years were lost due to cancer among most deprived patients, compared to the least deprived, although the age pattern of these inequalities varies according to cancer prognosis. The observations slightly differed when focussing on the proportion of the total LYL instead of their number.

**Fig. 4:**
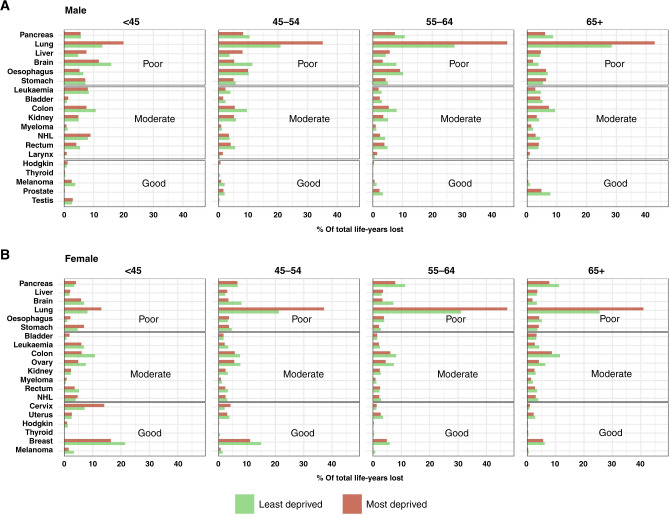
Proportion of number of life-years lost within 3 years due to the cancer indicated, in the most and the least deprived group, by age group and according to cancer prognosis (poor, moderate, good). **A** Male, **B** female; NHL non-hodgkin lymphoma.

Poor-prognosis cancers still accounted for the largest proportion of the total LYL for all cancers regardless of age and deprivation. However, figures can vary widely by deprivation. For example, in the most deprived, lung cancer contribution ranges from 13% (young female) to over 40% in age group 65+ (both sexes) (Fig. 4), while lung cancer represents only 21% of all incident cancers included in this deprivation group (Table 1). In the least deprived, the highest lung cancer contribution remains below 30% of LYL (65+ male) (Fig. 4) while 10% of cancers are from lung in this group (Table 1). Lung cancer remains the largest contributor of NLYL in all age groups, with the exception of female patients, aged 15–44 years, for whom the largest contributors of NLYL were breast cancer (least and most deprived) and cervical cancer (most deprived) (Fig. 4). A few cancer sites, such as brain, bowel, leukaemia, ovary and breast, are larger contributors of LYL in the least deprived than in the most deprived groups.

## Discussion

Our study, including the additional online infographic, clearly show that more deprived patients systematically lose more lifetime due to cancer, and that most deprived patients tend to stand out from the other deprivation categories with generally much higher NLYL. Those living in the most socioeconomically deprived neighbourhoods in England, accounting for around 17% of the incident cancers included in this study, lost 1.5 times more NLYL than the least deprived (0.98 years vs. 0.67 within 3 years; results not shown). To obtain these results, we used a relative survival approach, which allows the competing risks of death from other causes to be controlled without any information on the cause of death. Overall, the burden of poor-prognosis cancers is the highest, both regarding the NLYL and their proportions.

The largest socio-economic inequalities in NLYL were seen mostly in younger adults less than 45 years diagnosed with poor-prognosis cancers whilst for the moderate/good prognosis cancers the socio-economic inequalities varied substantially but with an overall widening, counterintuitive, trend with increasing age. The disproportionate socio-economic inequalities in younger adults were more specifically seen for the cancers related to tobacco smoking, such as pancreatic, lung and oesophageal cancers which presented the largest gaps in this age group. The prognosis of these cancers is so poor in older patients that survival differences can no longer be observed. In contrast, the narrow socio-economic inequalities from the good prognosis cancers, particularly among young patients, may be due to the ‘ceiling effect’, when survival in the less deprived is so high that it cannot improve further [4, 10].

Pancreatic cancer illustrates well this age-related pattern. In the age group less than 45 years, the most deprived male patients lost about 5 months more than the least deprived within 3 years, while in the age group 65 plus, this difference is only about 1 month. This is more likely due to very low survival probabilities, rather than reduced inequalities in the oldest age group. Five-year net survival from pancreatic cancer in England ranges between 36% in patients less than 45 years and 3% in those over 75 [45], which makes it almost impossible to detect any differences in this age group. The lack of early symptoms and advanced stage at diagnosis dramatically affect the probability of receiving surgical resection which is the only curative treatment for pancreatic cancer [46].

Similar phenomenon combined with lower use of a potentially curative treatment particularly in young deprived patients could explain the larger deprivation inequalities observed for lung cancer in younger patients. Surgical resection remains the major potentially curative treatment of lung cancer (particularly non-small-cell carcinoma). The receipt of surgical treatment decreases dramatically with age and deprivation, even after accounting for comorbidity [24], which is less of a concern among younger patients because of low comorbidity prevalence [47]. With the exceptions of youngest females and youngest least deprived males, lung cancer is also the largest contributor to LYL (Fig. 4). In the most deprived group, lung cancer represents a fifth of the incident cases (Table 1) and accounts for around 13–42% of all NLYL from all cancers combined, depending on sex and age. This highlights that a targeted lung cancer screening is justifiable given a large number of LYL that could be avoided [48, 49].

In addition to the aforementioned cancers, the largest socio-economic inequalities in NLYL overall were also seen for bladder cancer in young female patients and for laryngeal cancer in young male patients, both cancers related to tobacco smoking. Bladder [50] and colon [51] cancer cases illustrate the persisting gender inequalities in diagnosis, with early symptoms such as haematuria and pelvic pain less prone to further diagnostic investigations among women [51]. These inequalities are probably exacerbated among more deprived patients, who may not get access to a specialised healthcare facility for urologic surgery, either because of greater travel distance or lack of social support [52]. Regarding laryngeal cancer, the large deprivation gap in LYL seen in young men is unlikely to be explained by late diagnosis (i.e., advanced stage) [53], and more likely by suboptimal care, such as delayed treatment [54], or because of the poorer ability of deprived patients in navigating the complex laryngeal cancer pathway [55].

Cervical cancer is another important contributor, especially in women younger than 45 years, where it accounts for 15% and 7% of all LYL in most and least deprived patients, respectively, illustrating the need for increasing the cervical cancer screening uptake and HPV vaccine coverage among young women, particularly in more deprived population.

The study findings highlight the fact that reducing inequalities in younger adults is equally as important as tackling inequalities in the older population as it would result in many life-years gained. From a societal aspect, the LYL due to cancer in adults of working age can have a significant societal and economic impact. Studies in the US and Europe have consistently shown that premature loss of life attributed to cancer, results in reduced productive capacity and therefore loss in labour force earnings [56–59]. In the UK, it was estimated that in a single year over 50,000 people of working age lose their lives from cancer and in 2014 these people could have contributed £585 million to the UK economy [60]. Loss in productivity can also affect cancer survivors, especially those with short survival cancers or other co-morbid chronic diseases [61]. It is estimated that among cancer survivors only around 63.5% will return to employment with the majority reducing the working hours and limiting voluntary activities and caregiving [62].

Literature on the societal and economic impact of socio-economic inequalities in cancer remains scarce [63, 64]. Moreover, similar studies on this topic have mostly used the loss in life expectancy, which requires extrapolation of cancer survival of the cohort individuals up to the end of their expected life [65]. Our metric of LYL does not rely on such extrapolation as it is time-bound to the point where all patients have been followed up. We acknowledge that the social and economic costs of a patient death go far beyond 3 years. However, our estimates bounded at 3 years make the costs easier to estimate by health economists and more usable politically and for health policy planning.

From a public health policy perspective, it is vital to address these inequalities as this will reduce the overall impact of cancer on society. The wider inequalities among young patients potentially emphasise the structural components that may play a key role and pose a serious challenge to the healthcare system and society. Moreover, the range of these across-cancer inequalities poses the question of their causes. Mechanisms underlying such inequalities within a universal health coverage setting are still not well understood [66].

In a context of an increasing shortage of resources in both primary and secondary care sectors [67], the COVID-19 pandemic has exacerbated the inequalities [68, 69]. It also emphasised that the suboptimal distribution of resources between areas according to their deprivation level [70, 71] is likely to play an important role in the inequalities in accessing optimal healthcare [72] and, ultimately, in cancer outcomes [73]. The inequities component should be systematically and carefully considered in any policies aiming at improving cancer outcomes (including for earlier detection or new treatment) before their implementation in order to reduce these inequalities or even avoid further widening.

## Supplementary information

Supplemetary material

Appendix

Reproducibility checklist

## Data Availability

The data used for this study are the English National Cancer Registry data 1971–2014. Cancer registration data consist of patient information and as such, it is protected under the Data Protection Act 1998 and GDPR 2018 and cannot be made available as open data. Formal requests for release of cancer registration data can be made to the data custodian Public Health England (PHE), Office for Data Release (ODR) at odr@phe.gov.uk. The researchers will have beforehand obtained all the ethical and statutory approvals required for accessing sensitive data. Detailed information on the application process can be found at https://www.gov.uk/government/publications/accessing-public-health-england-data/about-the-phe-odr-and-accessing-data.
