# Supplementary material for: Socio-economic inequalities in cancer survival: how do they translate into Number of Life-Years Lost?
Source: Br J Cancer. 2022 Feb 11;126(10):1490–8. doi: 10.1038/s41416-022-01720-x (PMC9090931; doi:10.1038/s41416-022-01720-x)
Supplement: Supplementary file 1 — Supplemetary material [file 41416_2022_1720_MOESM1_ESM.pdf]

Supplementary material

Socio-economic inequalities in cancer survival: how do they translate into Number of Life-Years Lost?

Authors

Aimilia Exarchakou[1], Dimitra-Kleio Kipourou[1], Aurélien Belot[1], Bernard Rachet[1]

Authors' Affiliation

[1] Inequalities in Cancer Outcomes Network (ICON), Department of Non-Communicable Disease Epidemiology, Faculty of Epidemiology and Population Health, London School of Hygiene and Tropical Medicine, Keppel Street, London WC1E 7HT, UK

Figure 1 Probability of death up to 3 years since diagnosis for all cancer sites in male and female patients diagnosed in 2010-2014; NHL=Non-Hodgkin Lymphoma

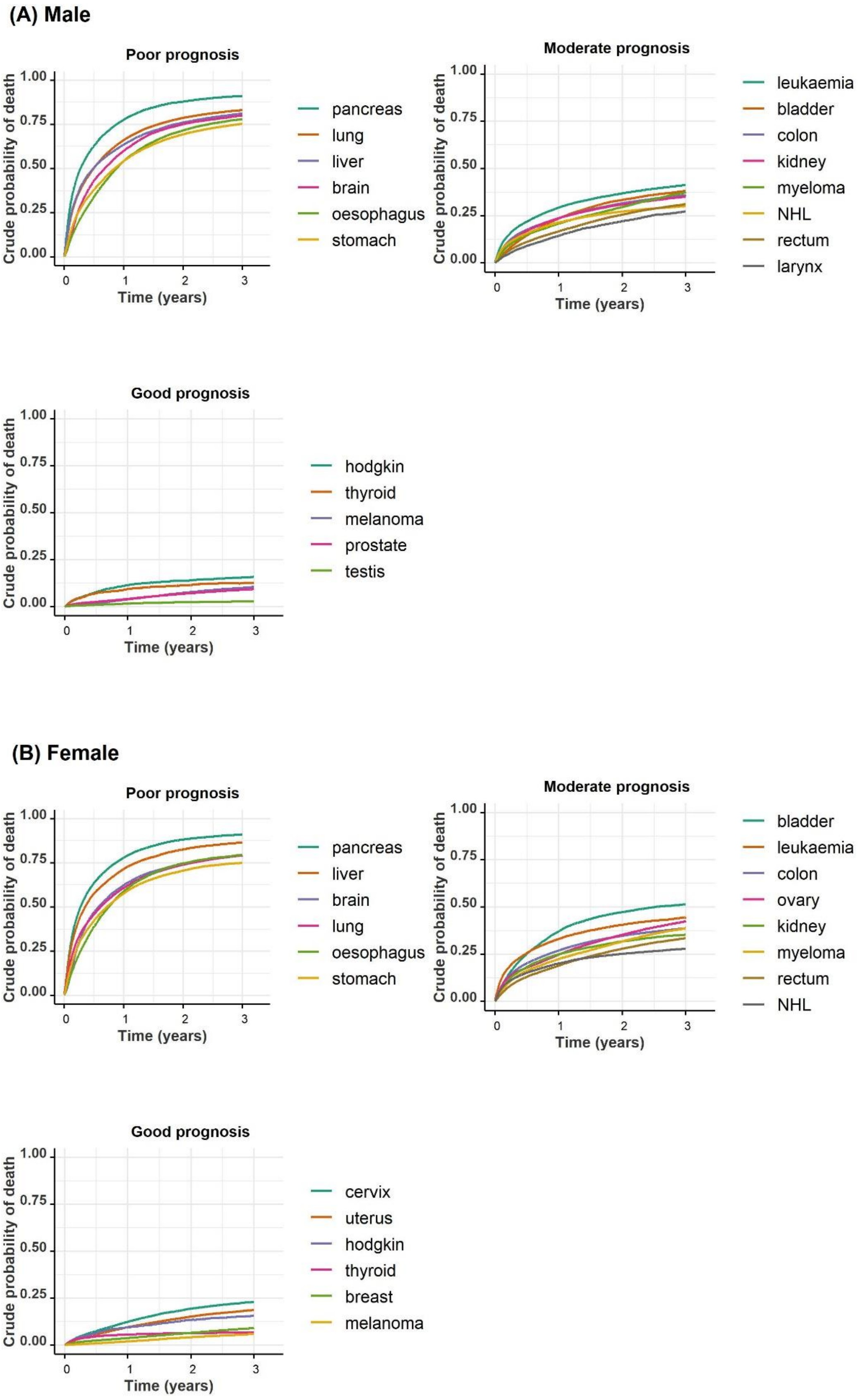

Table 1 Poor prognosis cancers: Number of Life-Years Lost within 3 years since cancer diagnosis in the least and most deprived patients

		Male				Female			
		Least deprived		Most deprived		Least deprived		Most deprived	
**Cancer**	**Age**	**Life-Years Lost**	**95% CI**	**Life-Years Lost**	**95% CI**	**Life-Years Lost**	**95%CI**	**Life-Years Lost**	**95% CI**
**brain**	**<45**	**0.68**	0.57 - 0.8	**0.78**	0.77 - 0.87	**0.47**	0.36 - 0.59	**0.66**	0.52 - 0.79
	**45-54**	**1.41**	1.29 - 1.52	**1.45**	1.39 - 1.61	**1.24**	1.08 - 1.38	**1.42**	1.24 - 1.6
	**55-64**	**1.83**	1.76 - 1.91	**1.98**	1.93 - 2.08	**1.82**	1.73 - 1.93	**1.88**	1.73 - 2.02
	**65plus**	**2.43**	2.39 - 2.47	**2.42**	2.38 - 2.47	**2.42**	2.38 - 2.47	**2.48**	2.42 - 2.54
**liver**	**<45**	**1.58**	1.19 - 1.96	**1.65**	1.53 - 1.84	**0.95**	0.58 - 1.38	**1.30**	0.95 - 1.68
	**45-54**	**1.33**	1.08 - 1.57	**1.73**	1.72 - 1.81	**1.45**	1.14 - 1.75	**1.59**	1.36 - 1.8
	**55-64**	**1.60**	1.49 - 1.73	**1.73**	1.69 - 1.8	**1.73**	1.55 - 1.89	**1.91**	1.76 - 2.04
	**65plus**	**2.07**	2.02 - 2.12	**2.11**	2.06 - 2.13	**2.21**	2.15 - 2.27	**2.28**	2.22 - 2.34
**lung**	**<45**	**1.41**	1.2 - 1.62	**1.72**	1.6 - 1.85	**0.95**	0.77 - 1.16	**1.49**	1.34 - 1.63
	**45-54**	**1.66**	1.55 - 1.76	**1.86**	1.8 - 1.92	**1.57**	1.47 - 1.67	**1.76**	1.7 - 1.82
	**55-64**	**1.84**	1.79 - 1.89	**1.94**	1.91 - 1.97	**1.61**	1.56 - 1.66	**1.73**	1.69 - 1.77
	**65plus**	**2.00**	1.98 - 2.02	**2.06**	2.04 - 2.07	**1.89**	1.86 - 1.91	**1.97**	1.95 - 1.99
**oesophagus**	**<45**	**1.41**	1.16 - 1.69	**1.64**	1.43 - 1.87	**1.12**	0.43 - 1.73	**1.72**	1.28 - 1.98
	**45-54**	**1.47**	1.34 - 1.6	**1.65**	1.55 - 1.75	**1.16**	0.94 - 1.36	**1.49**	1.3 - 1.66
	**55-64**	**1.48**	1.4 - 1.56	**1.64**	1.58 - 1.71	**1.34**	1.21 - 1.48	**1.56**	1.43 - 1.69
	**65plus**	**1.70**	1.66 - 1.74	**1.89**	1.85 - 1.93	**1.85**	1.8 - 1.9	**1.97**	1.91 - 2.02
**pancreas**	**<45**	**1.38**	1.05 - 1.71	**1.81**	1.56 - 2.07	**1.11**	0.77 - 1.43	**1.26**	1.02 - 1.51
	**45-54**	**1.93**	1.79 - 2.08	**2.04**	1.92 - 2.16	**1.62**	1.44 - 1.8	**1.86**	1.69 - 1.99
	**55-64**	**2.07**	2 - 2.15	**2.23**	2.16 - 2.3	**2.08**	2 - 2.16	**2.24**	2.15 - 2.32
	**65plus**	**2.37**	2.34 - 2.4	**2.46**	2.43 - 2.5	**2.36**	2.33 - 2.39	**2.44**	2.41 - 2.47
**stomach**	**<45**	**1.43**	1.17 - 1.69	**1.53**	1.33 - 1.74	**1.51**	1.2 - 1.82	**1.62**	1.4 - 1.85
	**45-54**	**1.37**	1.19 - 1.53	**1.37**	1.23 - 1.52	**1.49**	1.27 - 1.7	**1.46**	1.29 - 1.64
	**55-64**	**1.47**	1.36 - 1.59	**1.47**	1.36 - 1.57	**1.43**	1.25 - 1.61	**1.42**	1.28 - 1.58
	**65plus**	**1.72**	1.67 - 1.77	**1.83**	1.79 - 1.88	**1.77**	1.7 - 1.83	**1.84**	1.77 - 1.9

Table 2 Moderate prognosis cancers: Number of Life-Years Lost within 3 years since cancer diagnosis in the least and most deprived patients

		Male				Female			
		Least deprived		Most deprived		Least deprived		Most deprived	
**Cancer**	**Age**	**Life-Years Lost**	**95% CI**	**Life-Years Lost**	**95% CI**	**Life-Years Lost**	**95%CI**	**Life-Years Lost**	**95% CI**
**bladder**	**<45**	**0.35**	0.12 - 0.62	**0.51**	0.3 - 0.72	**0.63**	0.16 - 1.15	**1.26**	0.89 - 1.65
	**45-54**	**0.44**	0.32 - 0.56	**0.44**	0.34 - 0.56	**0.73**	0.51 - 0.98	**1.08**	0.85 - 1.33
	**55-64**	**0.52**	0.45 - 0.59	**0.62**	0.55 - 0.7	**0.73**	0.59 - 0.9	**0.94**	0.79 - 1.08
	**65plus**	**0.76**	0.73 - 0.79	**0.93**	0.89 - 0.97	**1.10**	1.04 - 1.16	**1.35**	1.28 - 1.41
**colon**	**<45**	**0.48**	0.38 - 0.58	**0.55**	0.44 - 0.64	**0.43**	0.35 - 0.52	**0.44**	0.35 - 0.52
	**45-54**	**0.58**	0.51 - 0.65	**0.70**	0.61 - 0.79	**0.44**	0.37 - 0.51	**0.65**	0.56 - 0.74
	**55-64**	**0.49**	0.45 - 0.53	**0.69**	0.64 - 0.75	**0.47**	0.43 - 0.52	**0.70**	0.63 - 0.76
	**65plus**	**0.79**	0.76 - 0.82	**0.97**	0.94 - 1	**0.87**	0.84 - 0.89	**1.09**	1.05 - 1.13
**kidney**	**<45**	**0.34**	0.22 - 0.47	**0.43**	0.33 - 0.53	**0.33**	0.17 - 0.51	**0.33**	0.21 - 0.45
	**45-54**	**0.42**	0.35 - 0.5	**0.58**	0.5 - 0.65	**0.44**	0.34 - 0.56	**0.44**	0.34 - 0.53
	**55-64**	**0.55**	0.49 - 0.61	**0.68**	0.61 - 0.74	**0.48**	0.4 - 0.56	**0.54**	0.47 - 0.63
	**65plus**	**0.83**	0.79 - 0.87	**1.01**	0.95 - 1.06	**0.87**	0.81 - 0.92	**1.02**	0.96 - 1.08
**leukaemia**	**<45**	**0.39**	0.29 - 0.48	**0.52**	0.43 - 0.61	**0.42**	0.31 - 0.53	**0.58**	0.46 - 0.7
	**45-54**	**0.42**	0.33 - 0.52	**0.55**	0.43 - 0.65	**0.46**	0.35 - 0.57	**0.62**	0.49 - 0.76
	**55-64**	**0.49**	0.42 - 0.56	**0.62**	0.53 - 0.7	**0.54**	0.46 - 0.64	**0.86**	0.74 - 0.99
	**65plus**	**1.12**	1.08 - 1.17	**1.16**	1.1 - 1.23	**1.23**	1.17 - 1.29	**1.34**	1.26 - 1.41
**larynx**	**<45**	**0.14**	0 - 0.48	**0.43**	0.21 - 0.66				
	**45-54**	**0.27**	0.12 - 0.46	**0.42**	0.31 - 0.54				
	**55-64**	**0.38**	0.27 - 0.5	**0.52**	0.44 - 0.59				
	**65plus**	**0.51**	0.43 - 0.59	**0.66**	0.59 - 0.74				
**myeloma**	**<45**	**0.36**	0.14 - 0.61	**0.29**	0.13 - 0.49	**0.18**	0 - 0.42	**0.32**	0.14 - 0.51
	**45-54**	**0.22**	0.12 - 0.32	**0.36**	0.25 - 0.47	**0.31**	0.17 - 0.44	**0.37**	0.23 - 0.54
	**55-64**	**0.32**	0.26 - 0.4	**0.55**	0.45 - 0.66	**0.30**	0.22 - 0.38	**0.45**	0.33 - 0.58
	**65plus**	**0.79**	0.74 - 0.85	**0.92**	0.85 - 0.99	**0.85**	0.8 - 0.91	**0.96**	0.89 - 1.04
**Non-Hodgkin Lymphoma**	**<45**	**0.26**	0.19 - 0.34	**0.37**	0.3 - 0.44	**0.17**	0.11 - 0.24	**0.32**	0.25 - 0.41
	**45-54**	**0.23**	0.17 - 0.29	**0.45**	0.37 - 0.52	**0.24**	0.18 - 0.31	**0.37**	0.29 - 0.46
	**55-64**	**0.41**	0.36 - 0.46	**0.61**	0.53 - 0.68	**0.29**	0.24 - 0.34	**0.47**	0.4 - 0.54
	**65plus**	**0.78**	0.74 - 0.81	**1.02**	0.96 - 1.07	**0.72**	0.68 - 0.76	**0.94**	0.88 - 0.99
**rectum**	**<45**	**0.45**	0.32 - 0.58	**0.51**	0.39 - 0.63	**0.45**	0.31 - 0.58	**0.59**	0.45 - 0.73
	**45-54**	**0.36**	0.3 - 0.43	**0.56**	0.49 - 0.64	**0.28**	0.21 - 0.35	**0.45**	0.36 - 0.55
	**55-64**	**0.38**	0.34 - 0.42	**0.57**	0.52 - 0.62	**0.27**	0.23 - 0.32	**0.57**	0.49 - 0.66
	**65plus**	**0.59**	0.56 - 0.62	**0.84**	0.8 - 0.88	**0.71**	0.67 - 0.75	**0.93**	0.87 - 0.98

Table 3 Good prognosis cancers: Number of Life-Years Lost within 3 years since cancer diagnosis in the least and most deprived patients

		Male				Female			
		Least deprived		Most deprived		Least deprived		Most deprived	
Cancer	Age	Life-Years Lost	95% CI	Life-Years Lost	95% CI	Life-Years Lost	95%CI	Life-Years Lost	95% CI
**Hodgkin Lymphoma**	**<45**	**0.04**	0.01 - 0.07	**0.06**	0.03 - 0.1	**0.05**	0.02 - 0.09	**0.07**	0.04 - 0.11
	**45-54**	**0.10**	0.01 - 0.19	**0.36**	0.21 - 0.51	**0.05**	0.01 - 0.13	**0.17**	0.05 - 0.33
	**55-64**	**0.21**	0.08 - 0.36	**0.60**	0.43 - 0.79	**0.24**	0.07 - 0.44	**0.60**	0.35 - 0.85
	**65plus**	**1.05**	0.87 - 1.22	**1.07**	0.86 - 1.3	**0.95**	0.76 - 1.14	**0.90**	0.68 - 1.11
**thyroid**	**<45**	**0.04**	0 - 0.09	**0.04**	0 - 0.08	**0.00**	0 - 0.01	**0.00**	0 - 0.01
	**45-54**	**0.15**	0.06 - 0.24	**0.06**	0.01 - 0.14	**0.04**	0.01 - 0.07	**0.02**	0.01 - 0.06
	**55-64**	**0.19**	0.09 - 0.3	**0.19**	0.08 - 0.33	**0.11**	0.07 - 0.17	**0.11**	0.04 - 0.2
	**65plus**	**0.58**	0.45 - 0.7	**1.01**	0.78 - 1.26	**0.53**	0.43 - 0.63	**0.62**	0.48 - 0.76
**skin melanoma**	**<45**	**0.06**	0.04 - 0.09	**0.14**	0.09 - 0.19	**0.04**	0.02 - 0.05	**0.04**	0.03 - 0.06
	**45-54**	**0.08**	0.05 - 0.1	**0.16**	0.1 - 0.21	**0.04**	0.02 - 0.05	**0.08**	0.05 - 0.12
	**55-64**	**0.10**	0.08 - 0.13	**0.26**	0.19 - 0.32	**0.05**	0.03 - 0.07	**0.07**	0.03 - 0.11
	**65plus**	**0.17**	0.15 - 0.19	**0.28**	0.23 - 0.34	**0.10**	0.08 - 0.12	**0.20**	0.14 - 0.25
**prostate**	**<45**	**0.03**	0 - 0.1	**0.08**	0.01 - 0.18				
	**45-54**	**0.05**	0.03 - 0.06	**0.10**	0.07 - 0.13				
	**55-64**	**0.05**	0.04 - 0.05	**0.09**	0.07 - 0.1				
	**65plus**	**0.18**	0.17 - 0.19	**0.22**	0.21 - 0.24				
**testis**	**<45**	**0.03**	0.02 - 0.05	**0.05**	0.04 - 0.07				
	**45-54**	**0.02**	0 - 0.04	**0.11**	0.04 - 0.18				
	**55-64**	**0.08**	0 - 0.17	**0.23**	0.08 - 0.42				
	**65plus**	**0.24**	0.07 - 0.44	**0.60**	0.25 - 1				
**breast**	**<45**					**0.08**	0.07 - 0.09	**0.12**	0.11 - 0.14
	**45-54**					**0.05**	0.04 - 0.06	**0.10**	0.09 - 0.12
	**55-64**					**0.05**	0.05 - 0.06	**0.11**	0.09 - 0.12
	**65plus**					**0.19**	0.18 - 0.2	**0.31**	0.29 - 0.33
**cervix**	**<45**					**0.12**	0.09 - 0.15	**0.22**	0.19 - 0.25
	**45-54**					**0.27**	0.19 - 0.35	**0.49**	0.41 - 0.56
	**55-64**					**0.52**	0.39 - 0.64	**0.50**	0.4 - 0.6
	**65plus**					**1.19**	1.07 - 1.32	**1.19**	1.08 - 1.3
**ovary**	**<45**					**0.22**	0.17 - 0.29	**0.17**	0.13 - 0.2
	**45-54**					**0.32**	0.27 - 0.37	**0.43**	0.37 - 0.5
	**55-64**					**0.54**	0.49 - 0.6	**0.66**	0.6 - 0.73
	**65plus**					**1.16**	1.12 - 1.2	**1.36**	1.3 - 1.42
**uterus**	**<45**					**0.23**	0.13 - 0.35	**0.25**	0.16 - 0.33
	**45-54**					**0.18**	0.14 - 0.22	**0.23**	0.18 - 0.28
	**55-64**					**0.17**	0.14 - 0.2	**0.23**	0.19 - 0.27
	**65plus**					**0.40**	0.37 - 0.43	**0.53**	0.49 - 0.58
