# Supplementary material for: Socio-economic inequalities in cancer survival: how do they translate into Number of Life-Years Lost?
Source: Br J Cancer. 2022 Feb 11;126(10):1490–8. doi: 10.1038/s41416-022-01720-x (PMC9090931; doi:10.1038/s41416-022-01720-x)
Supplement: Supplementary file 2 — Appendix [file 41416_2022_1720_MOESM2_ESM.pdf]

Appendix for the paper entitled:

Socio-economic inequalities in cancer survival: how do they translate into Number of Life-Years Lost?

Authors

Aimilia Exarchakou[1], Dimitra-Kleio Kipourou[1], Aurélien Belot[1], Bernard Rachet[1]

Authors' Affiliation

[1] Inequalities in Cancer Outcomes Network (ICON), Department of Non-Communicable Disease Epidemiology, Faculty of Epidemiology and Population Health, London School of Hygiene and Tropical Medicine, Keppel Street, London WC1E 7HT, UK

Non-parametric estimation of Number of Life-Years Lost due to cancer

It has been shown that the integral of the cause-specific cumulative probability function until time *t* can be interpreted as the expected number of life-years lost due to that cause before time t. [1, 2]

In a cohort of cancer patients, the Number of Life-Years Lost (NLYL) can be split into NLYL due to cancer and NLYL due to other causes. NLYL due to cancer at time *t* can be estimated as a function of the cancer-specific cumulative incidence function F_C from time *0* to time *t* (*aka* the crude probability of death due to cancer):

$$L_C(0,t) = \int_0^t F_C(u)du \quad (1)$$

F_C is a continuous function which reflects the probability of dying from cancer before or at time t, in the presence of competing causes of death. NLYLs are estimated within a pre-specified time window [0,*t*] to avoid extrapolation in the estimation of the cancer-specific cumulative incidence function F_C beyond the maximum time when all patients of the cohort have been followed up.[3]

On a group level, F_C can be defined as:

$$F_C(t) = \int_0^t S(u)\, \mathrm{d}\Lambda_C\,(u) \quad (2)$$

where $S(u)$ is the all-cause survival at time u and $d\Lambda_C(u)$ is the increase in the cancer-specific cumulative hazard from time *0* to time *t* on the whole population ("marginal").

In the relative survival framework of methods, F_C is often referred to as Crude Probability of Death (CPr) and its estimation is based on the main assumption of this framework[3], that the overall mortality hazard $\lambda_{O_i}(t)$ of an individual can be expressed as the sum of the excess (i.e. cancer-specific) hazard $\lambda_{C_i}(t)$ and the hazard of death from other causes (i.e. population hazard)[4] $\lambda_{P_i}(t)$:

$$\lambda_{O_i}(t) = \lambda_{C_i}(t) + \lambda_{P_i}(t) \quad (3)$$

After combining these individual hazards, the estimation of the marginal $\mathrm{d}\Lambda_c(u)$ in **(2)** can be based on the difference between the marginal overall cumulative hazard and the marginal population (or expected) cumulative hazard:

$$\mathrm{d}\Lambda_C(u) = \mathrm{d}\Lambda_O(u) - \mathrm{d}\Lambda_P(u) \quad (4)$$

More details can be found in Perme *et al*[2, 4] and in section 2.3.1 from Kipourou *et al*.[5]

References

1. Andersen PK. Decomposition of number of life years lost according to causes of death. Stat Med. 2013;32:5278-85.
2. Perme MP, Pavlic K. Nonparametric Relative Survival Analysis with the R Package relsurv. J Stat Softw. 2018;87:1–27.
3. Belot A, Ndiaye A, Luque-Fernandez MA, Kipourou DK, Maringe C, Rubio FJ, et al. Summarizing and communicating on survival data according to the audience: a tutorial on different measures illustrated with population-based cancer registry data. Clin Epidemiol. 2019;11:53–65.
4. Perme MP, Stare J, Esteve J. On estimation in relative survival. Biometrics. 2012;68:113-20.
5. Kipourou DK, Perme MP, Rachet B, Belot A. Direct modeling of the crude probability of cancer death and the number of life years lost due to cancer without the need of cause of death: a pseudo-observation approach in the relative survival setting. Biostatistics. 2020:kxaa017.
